# Global profile of individuals undergoing total knee replacement through the PROGRESS-PLUS equity lens: Protocol for a systematic review

**DOI:** 10.4102/sajp.v78i1.1649

**Published:** 2022-04-20

**Authors:** Marisa Coetzee, Amanda M. Clifford, Jacobus D. Jordaan, Quinette A. Louw

**Affiliations:** 1 Division of Physiotherapy, Department of Health and Rehabilitation Sciences, Faculty of Medicine and Health Sciences, Stellenbosch University, Cape Town, South Africa; 2 Health Research Institute, Ageing Research Centre, School of Allied Health, University of Limerick, Limerick, Ireland; 3 Department of Orthopaedic Surgery, Faculty of Medicine and Health Sciences, Stellenbosch University, Cape Town, South Africa

**Keywords:** profile, knee replacement, PROGRESS-PLUS, health-equity, osteoarthritis, risk factors

## Abstract

**Background:**

Osteoarthritis (OA) is a heterogenous degenerative disorder often causing destructive joint changes with severe pain and functional disability. Modifiable and non-modifiable risk factors, social context and psychological factors influence the development and progression of the disease. Total knee replacement (TKR) aims at reducing pain and improving function and is more successful with pre-operative and post-operative rehabilitation. However, most international research on rehabilitation interventions is conducted in high income contexts.

**Objective:**

The aim of our systematic review is to gain an overview of the demographic and social profiles of adults undergoing TKR for primary knee OA in lower, middle- and high-income countries through a health equity lens to inform the translation of intervention research in local contexts.

**Methods:**

A systematic review will be conducted and reported according to the Preferred Reporting Items for Systematic Reviews and Meta-Analysis (PRISMA) statement. Eligibility criteria include observational studies and grey literature (theses) since the beginning of the databases reporting on demographic data of adults awaiting or undergoing TKR surgery. The PROGRESS-Plus framework will be used to describe equity elements.

**Results:**

A narrative summary and description of the global profile of individuals undergoing total knee replacement for osteoarthritis.

**Conclusion:**

A snapshot of the global demographic and social profile of individuals receiving TKR for primary knee OA through an equity lens will shed light on the similarities and differences between individuals from different contexts. Global demographic profile information may inform or assist in the development of translational strategies for evidence-based rehabilitation.

**Clinical implications:**

Translation of existing rehabilitation interventions to local contexts could improve pre-operative and post-operative outcomes for individuals on our surgical waiting lists.

## Introduction

Osteoarthritis (OA) is a degenerative chronic disease affecting ageing individuals. A 48% increase in disease prevalence has been reported over the past three decades (1990–2019) (Hunter, March & Chew 2020). This increase in occurrence and progression of OA is attributed to a global ageing population and rising obesity (Palazzo et al. 2016). As OA progresses, individuals often experience chronic pain accompanied by functional disability, which negatively affects their quality of life, their ability to work and the fulfillment of life roles (Kloppenburg & Berenbaum 2020). Increased healthcare utilisation, surgical intervention and loss of productivity because of OA have a major effect on the economy being at least twice the cost of non-OA individuals (Xie et al. 2016).

A global increase in years lived with disability (YLD) for OA was documented by the 2017 Global Burden of Disease (GBD) study, showing that OA accounted for 7.1% of the global musculoskeletal (MSK) burden (Hay et al. 2017; Kloppenburg & Berenbaum 2020). In addition, less-developed countries are ageing at a more rapid rate than more-developed countries, with the highest increase in the burden of MSK disorders being currently reported amongst low- and middle-income countries (LMICs) (Blyth et al. 2019; Hay et al. 2017). A review by Yahaya (2021) showed a high prevalence of OA in LMICs with one in six individuals living with OA. The increase in OA within LMICs may be affected by the health inequalities which affect the health status, access and opportunities of these populations (Yahaya et al. 2021).

According to evidence-based guidelines, conservative management strategies for OA include education, exercise and weight management (if needed) as well as appropriate and timely pharmacological intervention (Bannuru et al. 2019; Kolasinski et al. 2020; National Clinical Guideline Centre 2014; Rausch Osthoff, et al. 2018; The Royal Australian College of General Practitioners 2018). With more advanced disease progression, and when conservative management fails to provide the individual with effective pain relief, joint replacement surgery is advised (Bannuru et al. 2019; Jette et al. 2020; Kolasinski et al. 2020). Because of the elective nature of this surgery, individuals are often placed on a waiting list. The waiting time for joint surgery could range from 4 to 18 months, with some countries having waiting times of up to 9 years (Cronström et al. 2020; Desmeules et al. 2009; Johnson, Horwood & Gooberman-Hill 2014; Tsui & Fong 2018).

Prolonged waiting times for joint replacement surgery can lead to further progression of OA, increased pain, anxiety, deterioration in function and a further reduction in quality of life (Desmeules et al. 2009; Scott, MacDonald & Howie 2019). These effects are shown to be amplified in women from lower socio-economic backgrounds (Ackerman et al. 2005).

The knee is the most affected joint in the body with a prevalence rate of 22% for individuals aged over 40 years (Cui et al. 2020). The diagnosis of knee OA typically falls within two distinct categories, namely primary and secondary OA. Primary OA is when articular degeneration has no clear underlying reason compared with secondary OA that is linked to joint injury (previous fractures, ligament and meniscus injuries) as well as inflammatory conditions such as rheumatoid arthritis (RA) (Hsu & Siwiec 2021). Risk factors for the development of primary knee OA include age, gender, genetics, increased BMI, physical activity levels and occupational demands (Silverwood et al. 2015). In addition, risk factors impacting the clinical progression (i.e. level of pain experienced and functional ability) and structural progression of knee OA include socio-economic variables (i.e. level of education, social class), psychological factors (i.e. coping strategies, anxiety and depression) and the presence of comorbidities (Bastick et al. 2016; Deveza et al. 2017). Most of these risk factors are linked to the social context of the individual and alongside the personal factors, community perceptions and psychological influences have an impact on the health-related outcomes of individuals with OA (Luong et al. 2012). Therefore, an additional aspect in the management of individuals with primary knee OA should be the identification of modifiable risk factors, targeted with an appropriate intervention (Georgiev & Angelov 2019).

Considering the complex and heterogeneous presentation of people with knee OA, management strategies should consider individuals from their biopsychosocial context. However, the data used to explore the risk factors, social determinants and clinical and structural progression trajectories are mainly from higher income countries and do not necessarily reflect the profile of individuals with knee OA in LMICs where a large portion of the population have a low socio-economic status (SES) (Dell’Isola et al. 2016; Dell’Isola & Steultjens 2018; Deveza et al. 2017; Luong et al. 2012). International OA management interventions are typically designed for the context of a study with populations from higher incomes and may not be generalisable or transferable to local contexts in LMICs because of the difference in health equity.

In order to adopt or design programmes that are suitable and tailored to a local context, the global demographic profile of individuals with OA must be studied through an equity lens, which will allow for the identification of the factors that influence the variations in health outcomes. The PROGRESS-Plus framework is an acronym for place of residence, race/ethnicity/culture/language, occupation, gender/sex, religion, education, SES, social capital, age, disability, sexual orientation and other vulnerable groups (Kavanagh, Oliver & Lorenc 2008). This framework was developed for the description and assessment of social determinants related to health equity within and between populations (Kavanagh et al. 2008). The benefit of using such a framework is the inclusion of most descriptors linked to the variability of health outcomes (O’Neill et al. 2014), which provide insight for translation of interventions into different contexts.

To our knowledge, there are no studies published, which describe the global demographic and social profile of individuals undergoing total knee replacement (TKR). Having a snapshot of the global demographic and social profile of individuals receiving TKR for primary knee OA through an equity lens such as the PROGRESS-Plus framework will shed light on the similarities and differences amongst individuals from different countries and may inform or assist in the translation of existing rehabilitation interventions to local contexts. Therefore, the aim of our systematic review is to describe the demographic and equity profiles of adults undergoing TKR for primary knee OA in lower-, middle- and high-income countries to inform the translation of research on interventions in lower-income contexts.

### Research question

What are the demographic and health equity profiles of adults undergoing knee TKR for primary OA in low-, middle- and high-income countries?

The review objectives will be to:

- Summarise and present the demographic and equity information (PROGRESS-Plus framework) of individuals undergoing primary TKR for primary OA.
- Describe the similarities and differences in demographics and equity factors amongst low-, middle- and high-income countries using the PROGRESS-Plus equity framework.

## Methods

Our systematic review was registered through the International Prospective Register of Systematic Reviews (PROSPERO) (Review No. 284634 on https://www.crd.york.ac.uk/prospero/) and will be conducted and reported according to the Preferred Reporting Items for Systematic Reviews and Meta-Analysis (PRISMA) statement (Page et al. 2021).

### Eligibility criteria

1. Population: All studies reporting demographic and social profile information on adults (> 18 years of age) awaiting or undergoing primary TKR of the tibiofemoral joint for primary OA will be considered. Primary OA is the chosen focus as the degeneration is not linked to trauma (which may lead to OA changes at a younger age) or disease which may provide a different profile because of these underlying reasons. Studies reporting pre-operative data (follow-up studies) on individuals who received total joint replacement for knee OA will also be considered.
2. Study design(s): Observational studies reporting demographic and social information will be included. This may include cross-sectional, cohort or case-control designs, but must report on the baseline pre-surgical information. Randomised controlled trials (RCTs) and systematic reviews will be excluded as the review is not aimed at effectiveness of interventions and strict inclusion criteria linked to intervention studies may not reflect the true population profile.
3. Condition specific information: Studies reporting on patients with end-stage or severe OA will be included. In addition, studies reporting on the profile of individuals with inflammatory arthritis such as RA or secondary post-traumatic knee OA (knee joint fractures or ligament injuries) will be excluded if no distinction is made between the information of individuals with primary knee OA. Authors of studies will be contacted if necessary to confirm information of cases where the type of OA is unclear.
4. Language: No language restrictions will be applied. Articles in foreign languages will be managed according to the strategies proposed by Walpole (2019).
5. Time period: Studies will be included from the inception of the databases until the end of the review period.
6. Data: The following information will be sought: Place of residence, race/ethnicity/culture/language, occupation, gender/sex, religion, education, SES, social capital, age, disability, sexual orientation and other vulnerable groups, BMI, quality of life, severity of OA and comorbidities.
7. Grey literature: Dissertations and theses submitted on ProQuest and SciELO will also be included.

Definition of terms: The following terminologies will be used for our review:

- **Undergoing TKR** refers to individuals awaiting TKR or those who have received a TKR, but baseline pre-surgical information is reported.
- **Country income group classification** will be sought from the World Bank list of income classification of economies (https://www.worldbank.org/en/home) which is based on the Atlas gross national income per capita (low income, lower-middle income, upper-middle income and high income).
- **Primary osteoarthritis** is articular degeneration without any apparent underlying reason (Hsu & Siwiec 2021).
- **Secondary osteoarthritis** is the consequence of either an abnormal concentration of force across the joint as with post-traumatic causes or abnormal articular cartilage, such as RA (Hsu & Siwiec 2021).

### Search strategy

In consultation with our faculty librarian, an initial search was conducted using the Stellenbosch University online library to identify databases hosting relevant peer-reviewed articles as well as grey literature such as theses. The following databases PubMed (Medline), Scopus (abstracts from Elsevier and other sources), Ebscohost (Africa wide, CINAHL, academic premier, Health Source Nursing), Web of Science (web of science core and SciELO) and ProQuest will be used for this review. Key terms for the proposed search were identified through searching PubMed, the Cochrane Library (knee OA related articles and reviews), author key words and indexed terms from relevant articles and are presented in Table 1. The search string was developed, and the proposed search strategies for each database are shown in Table 2.

**TABLE 1:** Proposed key search terms.

Variables	Key concepts		
	Knee osteoarthritis	Total knee arthroplasty	Profile
**Free text terms/natural language terms**	‘[K]nee osteoarthritis’; ‘knee OA’; KOA; arthritis; ‘osteoarthritis*’	‘[T]otal knee arthroplasty’; TKA; ‘Total joint arthroplasty’; TJA; TJR; TKR; arthroplasty; ‘knee replacement’	Profile*; demographic*; characteristic*; quality of life; epidemiology*; prevalence; ‘waiting list*’
**Controlled vocabulary terms/subject terms**	Knee osteoarthritis [MeSH]; ‘osteoarthritis’ [MeSH]; ‘Osteoarthritis, Knee/statistics and numerical data’ [Mesh]; ‘Osteoarthritis, Knee/surgery’ [Mesh]	Arthroplasty [MeSH]; ‘Arthroplasty, Replacement, Knee’ [Mesh]; ‘total knee Arthroplasty’ [MeSH];	‘Quality of Life’ [Mesh]

TKR, Total knee replacement.

**TABLE 2:** Proposed search strategy for databases.

Database	Search strategy
PubMed	(‘Arthroplasty, Replacement, Knee’ [Mesh] OR TJR[tiab] OR ‘total knee Arthroplasty’ [tiab] OR TKA[tiab] OR ‘total joint arthroplasty’ [tiab] OR TJA [tiab] OR ‘Knee Joint/surgery’ [Mesh] OR ‘knee replacement’ [tiab] OR TKR [tiab]) AND (‘osteoarthritis’ [MeSH] OR ‘Osteoarthritis, Knee/statistics and numerical data’ [Mesh] OR ‘Osteoarthritis, Knee/surgery’ [Mesh] OR ‘osteoarthritis*’ [Title/Abstract]) AND (‘quality of life’ [Title/Abstract] OR ‘Quality of Life’ [Mesh] OR profile*[tiab] OR epidemiology*[tiab] OR characteristic*[tiab] OR demographic*[tiab] OR prevalence[tiab] OR ‘waiting list*’)
Scopus	(INDEXTERMS (‘Arthroplasty, Replacement, Knee’) OR TITLE-ABS (‘total knee Arthroplasty’) OR TITLE-ABS (‘TKA’) OR TITLE-ABS (‘total joint arthroplasty’) OR TITLE-ABS (‘TJA’) OR INDEXTERMS (‘Knee Joint/surgery’) OR TITLE-ABS (‘knee replacement’) OR TITLE-ABS (knee AND arthroplasty)) AND (INDEXTERMS (‘osteoarthritis’) OR INDEXTERMS (‘Osteoarthritis, Knee/statistics and numerical data’) OR INDEXTERMS (‘Osteoarthritis, Knee/surgery’) OR TITLE-ABS (‘osteoarthritis*’)) AND (TITLE-ABS (‘quality of life’) OR INDEXTERMS (‘Quality of Life’) OR TITLE-ABS (‘profile*’) OR TITLE-ABS (‘epidemiology*’) OR TITLE-ABS (‘characteristic*’) OR TITLE-ABS (‘demographic*’) OR TITLE-ABS (‘prevalence’) OR TITLE-ABS (‘waiting list*’)) AND (LIMIT-TO (SRCTYPE, ‘j’)) AND (LIMIT-TO (SUBJAREA, ‘MEDI’) OR LIMIT-TO (SUBJAREA, ‘HEAL’) OR LIMIT-TO (SUBJAREA, ‘NURS’) OR LIMIT-TO (SUBJAREA, ‘MULT’) OR LIMIT-TO (SUBJAREA, ‘NEUR’))
Ebscohost	((MH ‘Arthroplasty, Replacement, Knee+’) OR (TI ‘total knee Arthroplasty’ OR AB ‘total knee Arthroplasty’) OR (TI TKA OR AB TKA) OR (TI ‘total joint arthroplasty’ OR AB ‘total joint arthroplasty’) OR (TI TJA OR AB TJA) OR (MH ‘Knee Joint/surgery+’) OR (TI ‘knee replacement’ OR AB ‘knee replacement’)) AND ((MH ‘osteoarthritis+’) OR (MH ‘Osteoarthritis, Knee/statistics and numerical data+’) OR (MH ‘Osteoarthritis, Knee/surgery+’) OR (TI osteoarthritis* OR AB osteoarthritis*)) AND ((TI ‘quality of life’ OR AB ‘quality of life’) OR (MH ‘Quality of Life+’) OR (TI profile* OR AB profile*) OR (TI epidemiology* OR AB epidemiology*) OR (TI characteristic* OR AB characteristic*) OR (TI demographic* OR AB demographic*) OR (TI prevalence OR AB prevalence) OR ‘waiting list*’)) Limiters – Human; Expanders – Apply equivalent subjects; Search modes – Boolean/Phrase
Web of Science	(‘Arthroplasty, Replacement, Knee’ OR ‘total knee Arthroplasty’ OR TKA OR ‘total joint arthroplasty’ OR TJA OR ‘Knee Joint/surgery’ OR ‘knee replacement’) AND (osteoarthritis OR ‘Osteoarthritis, Knee/statistics and numerical data’ OR ‘Osteoarthritis, Knee/surgery’ OR osteoarthritis*) AND (‘quality of life’ OR ‘Quality of Life’ OR profile* OR epidemiology* OR characteristic* OR demographic* OR prevalence OR ‘waiting list*’)
ProQuest	(AB(‘total knee Arthroplasty’ OR TKA OR ‘total joint arthroplasty’ OR TJA OR ‘Knee Joint/surgery’ OR ‘knee replacement’) OR SU(‘total knee arthroplasty’)) AND AB(osteoarthritis*) AND AB(‘Quality of Life’ OR profile* OR epidemiology* OR characteristic* OR demographic* OR prevalence OR ‘waiting list*’) Dissertations and thesis

TKR, Total knee replacement.

### Study selection and procedure

Following the search of the databases as per Table 2, the identified studies will be exported from the databases as comma-separated values (CSV) files containing titles and abstracts. The CSV files containing the study titles and abstracts will be imported into the Rayyan Intelligent Systematic Review web-based software (https://www.rayyan.ai/), where it will be scanned for duplicates by the software. The duplicates will be removed, and the remaining titles will be reviewed for the initial screening using the eligibility criteria. After the exclusion of articles based on the titles, the remaining articles will undergo a review of their abstracts for inclusion. Full-text versions of the articles included after the abstract review will be sought for further review and final inclusion. Two independent reviewers will review the titles, abstracts and full-text according to the eligibility criteria and a third reviewer will be consulted in the event of disagreement. Reporting of the review process will be done according to the PRISMA 2020 flow diagram for new systematic reviews which includes searches of databases and registers only (Page et al. 2021).

### Assessment of methodological quality

Methodological quality of the final list of included studies will be assessed using the appropriate study design appraisal tool from the Strengthening the Reporting of Observational Studies in Epidemiology (STROBE) checklist (Vandenbroucke et al. 2007; Von Elm et al. 2007). Two reviewers will conduct the quality assessment independently from each other. Reviewers will meet to discuss their results and any disagreement will be settled by a third independent person.

### Data extraction and management

In addition to the study characteristics (e.g. publication title, authors, date of publication, journal, aims and objectives, study design, study setting, sample size and results), the PROGRESS-PLUS framework will be used as a guide to extract further relevant information required for applying an equity lens and includes place of residence, race/ethnicity/culture/language, occupation, gender/sex, religion, education, SES, social capital, age, disability and other vulnerable groups (Kavanagh et al. 2008). Furthermore, quality of life scores, comorbidities and severity of OA will be extracted for descriptive purposes. Data will be extracted by the main reviewer into a Microsoft Excel spreadsheet for record-keeping and analysis. All quantitative data will be extracted with their point and interval estimates. The second reviewer will carry out an audit on the extracted data upon completion for quality control.

### Data synthesis

Basic synthesis of the data will be done using the descriptive statistical information available from the studies. Although some heterogeneity in the presentation of data is expected, we anticipate that most of the studies will report the demographic and social data using percentages, averages and ranges. In the case of categorised data, the reviewers will group the data according to the best fit for description. Narrative description of information (i.e. types of co-morbidities, social context and occupation) will be grouped according to similar themes which will be discussed and agreed upon amongst the reviewers. Missing information will be reported and if any data conversions are required, the process used will be described in the final report. An additional focus will be to compare the differences and similarities between patient populations alongside the characteristics of the studies and the study settings to provide contextual insight. All the reviewers will be involved in the data synthesis and analysis.

### Ethical considerations

Ethical clearance was obtained from the Human Research Ethics Committee from Stellenbosch University as an addendum to an existing approved PhD project, reference number: S20/11/315 (PhD).

## Results

A narrative summary and description of the extracted information will be presented as a published article upon completion of the review. Syntheses (and differences between LMICs and high-income countries [HICs]) will be presented using tables and graphs to describe the profiles of people who developed end-stage or severe knee OA and required TKR surgery.

## Discussion

Knee OA can cause severe pain and functional disability in ageing individuals, which reduces their quality of life (Kloppenburg & Berenbaum 2020). However, a clear heterogenous presentation of individuals with knee OA is found within studies and certain subsets of patients do not find relief from symptoms with proposed key conservative or surgical interventions (Dell’Isola & Steultjens 2018; Deveza et al. 2017). As our understanding of OA develops, management strategies are starting to focus more on the identification of individual risk factors and the effect of the social determinants of health on the development and progression of OA (Caneiro et al. 2020; Cui et al. 2020; Luong et al. 2012).

## Conclusion

In conclusion, our review will provide an overview of the demographic and social profiles of adults undergoing TKR for primary knee OA in low-, middle- and high-income countries through the PROGRESS-PLUS health equity lens. This will highlight the differences and similarities amongst individuals from different social contexts and inform translation of individualised interventions.
